# Circulating MicroRNAs as Biomarkers of Gestational Diabetes Mellitus: Updates and Perspectives

**DOI:** 10.1155/2018/6380463

**Published:** 2018-04-12

**Authors:** Elisa Guarino, Chiara Delli Poggi, Giuseppina Emanuela Grieco, Valeria Cenci, Elena Ceccarelli, Isabella Crisci, Guido Sebastiani, Francesco Dotta

**Affiliations:** ^1^UO Diabetologia, Azienda Ospedaliera Universitaria Senese, Siena, Italy; ^2^Department of Medicine, Surgery and Neurosciences, University of Siena, Siena, Italy; ^3^Fondazione Umberto di Mario, Toscana Life Sciences, Siena, Italy

## Abstract

Gestational diabetes mellitus (GDM) is defined as any degree of carbohydrate intolerance, with onset or first recognition during second or third trimester of gestation. It is estimated that approximately 7% of all pregnancies are complicated by GDM and that its prevalence is rising all over the world. Thus, the screening for abnormal glucose levels is generally recommended as a routine component of care for pregnant women. However, additional biomarkers are needed in order to predict the onset or accurately monitor the status of gestational diabetes. Recently, microRNAs, a class of small noncoding RNAs demonstrated to modulate gene expression, have been proven to be secreted by cells of origin and can be found in many biological fluids such as serum or plasma. Such feature renders microRNAs as optimal biomarkers and sensors of in situ tissue alterations. Furthermore, secretion of microRNAs via exosomes has been reported to contribute to tissue cross talk, thus potentially represents, if disrupted, a mechanistic cause of tissue/cell dysfunction in a specific disease. In this review, we summarized the recent findings on circulating microRNAs and gestational diabetes mellitus with particular focus on the potential use of microRNAs as putative biomarkers of disease as well as a potential cause of GDM complications and *β* cell dysfunction.

## 1. Introduction

Gestational diabetes mellitus (GDM) is defined as any degree of carbohydrate intolerance, with onset or first recognition during second or third trimester of gestation [1].

Insulin resistance physiologically increases during second and third trimester of pregnancy in order to guarantee proper nutrient supply for the fetus [2]: normally, a compensatory increase in insulin secretion maintains glucose homeostasis [3]. The inadequate *β* cell adaptation to peripheral insulin resistance is likely to be the main pathophysiological mechanism of glucose intolerance and hyperglycemia that characterize GDM [4].

It is estimated that approximately 7% of all pregnancies are complicated by GDM and that its prevalence is rising all over the world [5]. Thus, the screening for abnormal glucose levels is generally recommended as a routine care component for pregnant women [6].

Currently, the screening and diagnosis of GDM is accomplished by a one-step strategy (75 g OGTT at 24th–28th week of gestation) [7]: as a consequence, treatments cannot start before the late third trimester, which already presents a high risk of fetal morbidity and mortality. Therefore, an early screening in the first or second trimester of pregnancy could be important to promptly set up an adequate therapy which normalizes blood glucose levels [8], thereby reducing GDM adverse pregnancy outcomes. Furthermore, a careful evaluation of gestational diabetes risk factors, predisposing to the typical pregnancy alterations of glucose homeostasis, is needed in order to open the path for an earlier diagnosis. Although epidemiological studies on GDM risk factors are limited in number and biased by other potentially confounding risk factors and study population variables, several of them strongly emerged; indeed, well-established risk factors for GDM include ethnicity, a family history of type 2 diabetes (first-degree relatives affected by T2D), high BMI (obesity or excessive adiposity), advanced maternal age, parity and multiple pregnancies, previous fetal macrosomia (or history of poor obstetric outcomes), and a history of GDM [9, 10]. The association of GDM risk factors to novel potential early biomarkers may help in the prevention of GDM complications during pregnancy and of future metabolic health problem as well. Indeed, GDM not only increases the risk for maternal and fetal complications during pregnancy but also predisposes to long-term complications both in the mother and in the offspring [11]. Once GDM is diagnosed, the risk for type 2 diabetes mellitus (T2DM) and cardiovascular diseases (CVD) increases in the mother. Particularly, the risk of developing T2DM increases by sevenfold, with a cumulative incidence of 60% at 10 years from GDM diagnosis. The rate of T2DM onset increases rapidly after delivery, continuing to increase thereafter without signs of a plateau. Moreover, women with prior GDM have a significantly higher rate of obesity, hypertension, and metabolic syndrome, which, together with altered levels of circulating inflammatory markers, are important risk factors for CVD [12].

Recently, several studies have evaluated the expression of circulating microRNAs (plasma/serum) in diabetes [13]; microRNAs have been associated with the regulation of *β* cell mass and function and with the immune system homeostasis and certainly represent major players in the pathogenesis of this group of chronic metabolic diseases [14].

Deregulation of microRNA expression has also been associated with GDM; thus, these molecules could represent potential early diagnostic biomarkers, due to their high stability in body fluids and their accessibility from maternal blood throughout gestation [15]. Therefore, a deep understanding of microRNA functions could improve the knowledge on etiology and pathophysiology of GDM and of its complications.

In this review, we aim at providing an overview of recent advances in the characterization of extracellular (plasma/serum) microRNAs in GDM.

## 2. MicroRNA Biogenesis and Secretion

MicroRNAs are small noncoding ~19–24 nucleotide- (nt-) long RNA molecules that play an important role in the modulation of gene expression [16]. They were discovered in 1993 in *Caenorhabditis elegans* [17] but afterward have been identified in plants, in vertebrates, and in some viruses. The number of discovered microRNAs has progressively increased: each of these molecules can target and regulate multiple genes, whereas a single target gene can be regulated by several different microRNAs [18]. Therefore, it is now clear that microRNAs are involved in many biological processes and that their deregulation or dysfunction can contribute to several diseases, including GDM [19].

MicroRNA biogenesis, function, and secretion are complex events involving a series of molecular mechanisms, which are not yet fully understood. MicroRNA biogenesis starts with microRNA gene transcription by RNA polymerase II (RNA Pol II) or RNA polymerase III (RNA Pol III): the first transcribes intragenic microRNA genes alongside with their host genes [20]; the latter transcribes intergenic microRNAs with their own promoter [21]. The transcription generates a long primary sequence (primicroRNA) that is usually capped and polyadenylated and has one or more long hairpin structure. PrimicroRNAs are then cleaved by the ribonuclease III enzyme *Drosha* and its cofactor DGCR8, generating a long hairpin-structured premicroRNA of ~60–70 nucleotides. The following maturation step is performed outside the nucleus, so the generated premicroRNAs are transported by Exportin-5 into the cytoplasm, where they are further cleaved by RNase III enzyme *Dicer* to generate 22 nucleotide double-stranded RNA with overhangs, consisting in a guide strand and in a passenger strand. While the passenger strand is usually degraded and, therefore, less represented in terms of expression levels, the guide strand, which is normally the most thermodynamically stable, is loaded into Argonaute proteins 1–4 (Ago 1–4) to form RNA-induced silencing complex (RISC) [22]. Functionally, mature microRNAs guide the RISC complex to recognize target mRNAs (messenger RNA), thus inducing a negative regulation. Target recognition is determined by the complementarity between the 3′-untranslated region of mRNA and bases 2–8 of the microRNA (called *seed* sequence) [23].

MicroRNAs can regulate target mRNAs through multiple pathways: the pairing between miRNA and its target site can lead to the degradation of mRNA by endonucleolytic cleavage mediated by Argonaute proteins or by deadenylation of mRNA molecule; the RISC complex can also induce the translational repression of mRNA or stimulate the proteolysis of the nascent peptide; finally, miRNAs have also been shown to upregulate target expression under certain conditions [24]. Translational repression and mRNA decay/degradation are considered the main microRNA mechanisms responsible for the regulation of their target mRNAs: whether these two mechanisms act in parallel or sequentially is still not fully understood. Recent studies have demonstrated that translational repression precedes mRNA degradation but is not always followed by mRNA degradation: indeed, it has been observed that a microRNA-repressed mRNA can be translationally reactivated [23]. It has been hypothesized that approximately 60% of genes are regulated by microRNAs; thus, these molecules are critically involved in the regulation of many biological processes [25].

Despite their function as regulators of gene expression, recent studies have demonstrated that microRNAs are not exclusively intracellular but also extracellular, being present in a cell-free circulating form in many different biological fluids, including serum and plasma [26].

Since the discovery of extracellular microRNAs in 2008 [27], researchers identified multiple mechanisms of microRNA transport, derived from different cell secretion/release pathways [28]:

- Passive release after cell death (vesicle-free) of microRNAs associated with AGO proteins
- Passive secretion through apoptotic bodies containing components of apoptotic cells, including microRNAs
- Active secretion of microRNAs packaged into shedding vesicles and exosomes
- Active secretion of microRNAs coupled with high-density lipoproteins (HDL) or low-density lipoproteins (LDL)

The vehicular transport of microRNAs within small lipid vesicles and the association of microRNAs to protein complexes protect them against degradation by RNAses and by other RNA-degradation agents, rendering extracellular microRNAs very stable molecules [29]. Owing to their stability in biofluids, it has been suggested that microRNAs may represent a new form of cell-to-cell communication, both in physiological and in pathological conditions [30]. Furthermore, a putative use of circulating microRNAs as diagnostic, prognostic, and therapeutic biomarkers of many different diseases (including diabetes) has been widely reported, as they can be easily detected and measured in body fluids [31, 32].

## 3. Placental MicroRNAs in Healthy Pregnancy and GDM

Pregnancy represents an enormous stress for the maternal body and requires many physiological changes in order to guarantee a proper embryo/fetal growth [33]. Surely, a pivotal role in driving the maternal adaptations and/or deregulations is played by placenta-derived molecules, such as placental lactogen, growth hormone and tumor necrosis factor alpha, and by increased cortisol and progesterone levels [2]. Furthermore, additional novel molecules, such as placenta-derived microRNAs, have also been demonstrated to be involved in these regulations, thus suggesting that their alterations could be detrimental for maternal adaptation mechanisms [34].

Several studies reported that human placenta expresses more than 500 microRNAs, and only a part of them are also expressed in other tissues and organs [35]. Due to the importance of the placenta for healthy pregnancy, the characterization of placental microRNAs could be essential to understand the regulatory mechanisms of normal and complicated pregnancies. The group of placental microRNAs has been recently subdivided into (i) placenta-specific, (ii) placenta-associated, and (iii) placenta-derived circulating microRNAs [36].

Placenta-specific microRNAs are specifically or predominantly expressed in the placental tissues and are mainly encoded by three microRNA gene clusters: chromosome 19 microRNA cluster (C19MC), chromosome 14 microRNA cluster (C14MC), and miR-371–373 cluster [37, 38]. C19MC encodes for 58 mature microRNAs, expressed by trophoblasts in the early stages of pregnancy and afterwards by placental differentiated cells. C14MC encodes for 63 mature microRNAs and is highly expressed in trophoblasts. Finally, miR-371–373 cluster is exclusive to mammals and encodes for six mature microRNAs, mostly expressed by placental differentiated cells and embryonic stem cells [36]. All these placenta-specific microRNAs are linked to cell proliferation, apoptosis, migration, and angiogenesis in trophoblasts, that is, the critical processes needed for adequate placentation in early pregnancy [39].

Placenta-associated microRNAs are ubiquitously expressed in the placenta and in other tissues, with different expression profiles across pregnancy. Gu et al. have recently identified 191 differentially expressed microRNAs between placentas at first versus third trimester of gestational age: during the first trimester, oncogenic, angiogenic, and antiapoptotic microRNAs predominated, whereas during the third trimester, microRNAs related to cell differentiation and tumor suppression prevailed [36, 40]. Many studies demonstrated that both placenta-specific and placenta-associated microRNAs can be deregulated in pregnancy complications, such as pregnancy loss, preeclampsia, intrauterine growth restriction/fetal growth restriction, preterm birth, and GDM, suggesting their role in the pathogenesis of these conditions [41]. Several studies analyzed the expression of placenta microRNAs during disease conditions and highlighted GDM-specific deregulations of such molecules. One of the first studies in such direction uncovered placenta-specific microRNA miR-518d alteration in GDM. Indeed, microRNA miR-518d, one of the highest expressed C19MC placenta-specific microRNAs, was found to be hyperexpressed in placenta obtained from GDM patients with respect to nondiabetic women who delivered between the 37th and 40th week of gestation. The same research group uncovered a specific regulation of miR-518d on peroxisome proliferator-activated receptor-*α* (PPAR*α*) gene (associated with metabolic adaptations during pregnancy), thus modulating its expression; interestingly, placental expression of PPAR*α* is inversely correlated to miR-518d, thus further demonstrating the control of PPAR*α* expression by such microRNA during pregnancy [42].

A subsequent study performed a similar analysis by taking into consideration placental tissues obtained by GDM and non-GDM patients [43]. The microarray analysis revealed a set of deregulated microRNAs (miR-508-3p, miR-27a, miR-9, miR-137, miR-92a, miR-33a, miR-30d, miR-362-5p, and miR-502-5p) which, collectively, targets key genes involved in epidermal growth factor receptor (EGFR) signaling cascade, thus highlighting their potential involvement in the induction of macrosomia, a typical fetal GDM-related complication, strongly associated to the alteration of EGFR signaling [44].

Additional studies uncovered several other microRNAs whose expression is altered in placenta of GDM patients and potentially linked to its function or development. This is the case of miR-221 and miR-222, whose expression was found to be upregulated in human fetoplacental endothelial cells (fpEC) isolated from third-trimester human placentas after pregnancies complicated by GDM versus healthy pregnancies; importantly, miR-221 and miR-222 were found to target ICAM1 protein species (ICAM-1, V-CAM, and E-selectin), whose expression was reduced in GDM placentas. The authors pointed out this mechanism as a protection against leucocyte transmigration from blood to placenta which may worsen the inflammation due to hyperglycemia during GDM [45].

Finally, it has been reported that placenta-derived circulating microRNAs are released into maternal circulation mostly by syncytiotrophoblasts. They are carried in plasma by exosomes as early as the sixth week of gestation [46], even though other microRNA carriers (e.g., AGO proteins, HDL) cannot be excluded. These microRNAs reflect (at least partially) the expression of placenta-specific and placenta-associated microRNAs, mirroring physiological and pathological conditions during pregnancy. For this reason, many researchers have proposed and investigated their use as diagnostic biomarkers [41], as microRNAs can be easily detected and measured from the blood.

The first study demonstrating a specific case of placenta-blood mirroring in GDM and involving microRNAs has been recently published by Xu and colleagues. The authors identified the microRNA miR-503 which was upregulated both in the placenta and peripheral blood of GDM patients (*n* = 25) versus nondiabetic subjects (*n* = 25). Interestingly, the hyperexpression of miR-503 in rat *β* cell line INS-1 reduces the proliferation and insulin secretion while promoting apoptosis. Although such study needs specific validations and further experimental evidences, it opens to the possibility of a microRNA-based specific cross talk between placenta and *β* cells in gestational diabetes [47].

## 4. Circulating MicroRNAs as Candidate Biomarkers of Gestational Diabetes Mellitus

Recently, several studies have evaluated the expression of circulating microRNAs (plasma/serum) in diabetes, in order to establish whether microRNAs may represent early biomarkers of this group of chronic metabolic diseases and to clarify their eventual involvement in the pathogenetic mechanisms [13]. As a matter of fact, circulating microRNAs have been associated with *β* cell function and regulation as well as with immune system homeostasis, representing major players in diabetes pathogenesis [14, 48, 49], and deregulation of microRNA expression has been associated with metabolic disorders characterized by impaired insulin secretion and/or action [50].

Previous studies have been mainly focused on the expression of circulating microRNAs in T1D and T2D, while only few have investigated the expression and the diagnostic utility of circulating serum/plasma microRNAs in GDM (Table 1).

The first published study reporting an association between circulating microRNAs and GDM was performed by Zhao et al. in 2011. The authors evaluated microRNAs expression in sera obtained from GDM patients and non-GDM controls between the 16th and 19th week of pregnancy, with the aim to identify a microRNA signature potentially useful for an earlier diagnosis and prediction of GDM. Initially, by using TaqMan MicroRNA Array microfluidic cards, the authors analyzed a pool of 24 sera derived from GDM patients and a pool of 24 sera derived from nondiabetic subjects; differentially expressed microRNAs were further validated in another cohort of 36 GDM patients and 36 nondiabetic controls. They reported that three microRNAs (miR-29a, miR-132, and miR-222) were downregulated in serum of women affected by GDM versus nondiabetic subjects [51]. Despite such differential expression, the receiver operating characteristic (ROC) curves did not retrieve high sensitivity and specificity (combined microRNAs: sensitivity = 66.7% and specificity = 63.3%) to clearly distinguish between GDM and controls. Therefore, although a potential future use of these microRNAs as biomarkers of GDM requires further studies, their involvement in GDM pathogenic mechanisms could be of immediate interest. Indeed, the authors reported that, among the validated target genes of miR-29a, there is *Insig1*, an inhibitor of proteolytic activation of sterol regulatory element-binding proteins (SREBPs). This latter, in turn, activates genes involved in cholesterol and fatty acid metabolism and, probably, in glucose homeostasis, such as PCK2, a key enzyme in hepatic gluconeogenesis [52, 53]. Moreover, recent studies have demonstrated that both miR-29a and miR-222 directly and/or indirectly regulate the glucose transporter member 4 (GLUT4), which plays a key role in glycaemic control and in insulin-induced glucose uptake by muscle and adipose tissues; as a matter of fact, its expression/translocation is reduced in prediabetes and in diabetes [54]. Therefore, the hypothesis of a cross-talk effect mediated by circulating microRNAs and targeting insulin-sensitive tissues is of particular interest in order to understand the molecular cues underlying GDM pathogenesis.

Zhu et al. have evaluated microRNA expression in the plasma of 10 GDM patients and 10 non-GDM controls at 16th–19th week of pregnancy, using next-generation sequencing approach. With respect to the previous study, they identified a different microRNA signature composed of five microRNAs (miR-16-5p, miR-17-5p, miR-19a-3p, miR-19b-3p, and miR-20a-5p) which are upregulated in the plasma of women affected by GDM versus nondiabetic subjects. Such discrepancy could be attributed to the use of different starting sample (serum versus plasma) or differences in the use of the analysis platform. However, the microRNAs identified by Zhu et al. are reported as mainly associated with MAPK signaling pathway, insulin signaling pathway, TGF-*β* signaling pathway, and mTOR signaling pathway, which are all involved in insulin secretion [55].

In another recent study, Cao et al. tried to confirm the results of the pilot study by Zhu et al. in a larger group of patients (85 GDM women and 72 non-GDM women), by analyzing the same differentially expressed microRNAs (miR-16-5p, miR-17-5p, miR-19a-3p, miR-19b-3p, and miR-20a-5p) found by Zhu and colleagues. They did not find any significant differences in miR-19a-3p and miR-19b-3p expression between GDM and non-GDM patients, whereas miR-16-5p, miR-17-5p, and miR-20a-5p were confirmed as progressively upregulated during pregnancy in the plasma samples of GDM women at 16th–19th week, 20th–24th week, and 24th–28th week of pregnancy [56]. The role of these upregulated microRNAs in the pathogenesis of GDM is not yet fully understood, although some studies reported the involvement of miR-16-5p and of miR-17-5p in T2D and in other metabolic diseases. Interestingly, miR-16-5p target genes (*CUL4A*, *SMAD1*, *EGFR*, *ACTB*, *RRP12*, and *DAB2*) have been reported to be downregulated in T2D [57]; moreover, insulin receptor substrate (IRS) proteins 1 and 2, known as adaptor proteins that mediate insulin-like growth factor-1 (IGF-1) and insulin signaling in insulin-sensitive tissues (e.g., adipose tissue, bone, and liver) [58, 59] are reported as miR-16 target genes. In addition, IRS1 and IRS2 promote Wnt/*β*-catenin signaling that is critical for cell growth: it has been demonstrated that the dysregulation of this signaling pathway leads to cancer, obesity, and diabetes [60]. Although the confirmation of circulating miR-16-5p alteration in the plasma of GDM patients in two independent studies revealed a potential pivotal role for such microRNA in the pathophysiology of GDM, it is important to note the limited use of miR-16-5p as a potential biomarker; indeed, it has been demonstrated that miR-16-5p is highly expressed and enriched in erythrocytes, thus leading to the possibility of its expression level variation upon hemolysis of the sample; such characteristic renders potentially misleading the measurement of circulating miR-16-5p expression levels in order to monitor the development of GDM and, therefore, cannot be taken into consideration as a reliable biomarker.

Regarding miR-17-miR-20b microRNA family, their involvement in smooth muscle cell proliferation has been previously reported, potentially suggesting a specific role for these microRNAs in vascular complications in diabetic patients [61]. Furthermore, another study previously associated miR-17 and miR-20b to preeclampsia [62], a complication of pregnancy, which (i) affects perinatal outcomes, (ii) is highly correlated to GDM in terms of degree of glucose intolerance [63], and (iii) shares common risk factors with GDM [64]. It is not unlikely that different pregnancy complications (GDM, preeclampsia, etc.) may share similar circulating microRNA alterations due to the observed overlaps of these complications during pregnancy.

More recently, Wander et al. have evaluated the expression of 10 microRNAs (miR-126-3p, miR-155-5p, miR-21-3p, miR-146b-5p, miR-210-3p, miR-222-3p, miR-223-3p, miR-517-5p, miR-518a-3p, and miR-29a-3p), selected for their pivotal roles in pregnancy and its complications and/or previously associated to T2D, in a case-control prospective cohort study of pregnancy complications including plasma samples from 36 GDM patients and from 80 non-GDM controls collected during early–mid pregnancy (7th–23rd week of gestation). They found that high plasma levels of two microRNAs (miR-155-5p and miR-21-3p) were associated with GDM, while levels of other two microRNAs (miR-21-3p and miR-210-3p) were specifically associated with overweight/obese GDM women; finally, levels of six microRNAs (miR-155-5p, miR-21-3p, miR-146b-5p, miR-223-3p, miR-517-5p, and miR-29a-3p) were associated with GDM only among patients carrying male fetuses [65]. Previous studies have found an association of these microRNAs to the pathogenesis of T2D as well. For example, microRNA miR-29a was found upregulated in serum of patients with newly diagnosed or existing T2D [66] and regulates hepatic gluconeogenesis [51], insulin resistance in adipocytes cell lines [67], and GLUT4 expression [54]. Conversely, Zhao et al. have found miR-29a downregulated in serum of GDM patients, in contrast to the results of the previous study. Importantly, microRNAs associated with GDM observed by Wander et al. were limited to overweight/obese prepregnancy patients, probably due to the selection of candidate microRNAs analyzed, all involved in pathways that link obesity to T2D [65].

Finally, our group recently analyzed the expression profiles of plasma microRNAs in GDM patients versus nondiabetic subjects at 28th–33th week of gestation. Due to the high heterogeneity of the resulting differentially expressed microRNAs obtained in previous studies, we hypothesized that the preanalytical factors may strongly affect microRNA stability, leading to differences in their expression mainly due to different sampling methods and nonstandardization of blood collection and processing. Using a highly standardized approach, we identified the hyperexpression of miR-330-3p in the plasma of GDM patients versus nondiabetic subjects; furthermore, such microRNA expression levels were able to distinguish two subpopulations of GDM patients characterized by high and low miR-330-3p expression (miR-330-high, miR-330-low) and by differences in diabetic outcome. Moreover, as miR-330-3p targets key genes involved in *β* cell function (e.g., E2F1), we hypothesized that its plasma hyperexpression may be detrimental for *β* cell function and/or proliferation if transferred to them [68].

Although the alterations of circulating microRNAs in the plasma of GDM patients have been reported to be potentially associated to *β* cell dysfunction and impaired compensation, several authors started also to evaluate whether GDM microRNAs were associated to fetal abnormalities related to maternal diabetes. Fetal and neonatal neural system development alterations, resulting in intellectual and behavioral abnormalities, are strongly associated to GDM; although such association has been clearly established, the underlying mechanisms are less clear. Therefore, in order to verify whether microRNAs could be linked to such aspect of GDM-related complication, specific studies have been performed in such direction. To this aim, Lamadrid-Romero et al. evaluated the expression of 12 fetal neural development-related microRNAs (miR-183-5p, miR-200b-3p, miR-9-5p, miR-17-5p, miR-30b-5p, miR-30c-5p, miR-124-3p, miR-125b-5p, miR-128-3p, has-191-5p, miR-1290, and miR-137) in serum of nonpregnant healthy women and pregnant women (GDM and non-GDM) with the aim to find potential correlations between GDM and alterations of circulating microRNAs involved in fetal neural system development. The results showed that the levels of miR-193-5p, miR-200b-3p, and miR-125-5p were higher in the second trimester versus the first trimester of pregnancy, independently of GDM, while levels of miR-137 were higher in the third trimester in relation to the first trimester, revealing the time-related heterogeneity of these neural development-associated microRNAs. Furthermore, during the first trimester of pregnancy, GDM patients showed higher levels of miR-183-5p, miR-200b-3p, miR-125-5p, and miR-1290 versus controls, suggesting potential alterations of fetal neural differentiation and cell proliferation in this group of patients [69]. Interestingly, previous studies demonstrated that miR-183 and miR-200 gene families regulate cell proliferation in human glioblastoma cells [70, 71] and are involved in the equipoise between neuroepithelial proliferation and neuroblast emergence in the optic lobe of flies [72]. Furthermore, miR-200 can suppress the expression of Sox2 and reduce proliferation and multipotency of NSCs in the midbrain/hindbrain region in mice, thus strongly suggesting that its hyperexpression during GDM could impair fetal neural system development [73]. Collectively, the increased levels of these microRNAs in GDM patients during the first trimester, as reported by Lamadrid-Romero et al., suggest a decrease in cell proliferation and an increase in neuron differentiation during the development of fetal central nervous system [69], confirming the results of previous studies in mice [74, 75]. Finally, they suggest that neural development-associated microRNAs can be detected in the serum of pregnant women, potentially representing a mirroring of the physiological or pathological growth of the fetus during pregnancy. Indeed, it has been previously well established that fetal DNA and/or RNA may derive from fetal cell debris, and recent studies revealed that these molecules could have biological implications and may act as mediator of cell-to-cell communications between the mother and the fetus, both in physiological and in pathological conditions [36, 76]. However, whether such circulating microRNA alterations during GDM represent a cause of neural impairment or a consequence remains to be established, and further studies are needed in such context.

## 5. Exosomes and Gestational Diabetes Mellitus

In the last decade, several studies have focused on extracellular vesicles (EVs), classified as exosomes or microvesicles, according to their size, cell or tissue of origin, and functions [77].

Particularly, exosomes are bilayered lipid vesicles of 40–120 nm diameter and originate from the endosomal compartment by the fusion of multivesicular bodies with the plasma membrane of multiple cell types; they contain a wide range of molecules, such as RNA (including microRNAs) and proteins, and are involved in cell-to-cell communications by delivering their cargos into target cells. Therefore, exosomes play a key role in many biological processes and could be useful biomarkers of physiological and pathological conditions, as they can be isolated from body fluids (e.g., plasma, saliva, and urine) [29].

EVs and exosomes play several roles during pregnancy, from regulation of immune responses to maternal metabolic adaptation to gestation [78]. Interestingly, placenta can communicate with other organs/tissues and regulate maternal function via exosomes [79], whose secretion can be modulated by many extracellular stimuli, such as low oxygen tension or high glucose concentration [80, 81].

Importantly, it has been shown that the plasma concentration of specific placenta-derived exosomes is increased significantly with gestational age during first trimester in pregnant women compared to nonpregnant women [82, 83]; more specifically, such placenta-derived exosomes can be detected in maternal plasma at early gestation (~6 weeks) [46], and studies by Sarker et al. [83], Salomon et al. [84], and Nardi et al. [85] independently demonstrated that their concentration progressively increases across pregnancy and correlates with gestational age, while the release of specific trophoblast-derived exosomes decreases during the third trimester.

The first study, demonstrating the association of exosomes to GDM pathophysiology, was performed by Rice et al. [81], who demonstrated a significant increase of exosome concentration in the plasma of GDM pregnant women compared to non-GDM pregnant women and, furthermore, reported that high glucose concentration enhanced exosome release from first-trimester trophoblast cells. Salomon et al. [86] confirmed these results and further showed a differential release of proinflammatory cytokines from endothelial cells treated with placental exosomes from GDM women.

Additionally, Elfeky et al. [87] demonstrated that the total number of exosomes in maternal circulation was strongly correlated with maternal BMI, an important risk factor for GDM. A higher maternal BMI was also correlated with a decreased concentration of placental exosomes with respect to the total exosomal population and with an increased release of IL-6, IL-8, and TNF-*α* from endothelial cells, showing a possible contribution of exosomes to the maternal systemic inflammation during pregnancy [87].

Surely, further studies are required to elucidate the role of exosomes in GDM pathogenesis. In addition, considering that the deregulation of microRNA expression has been associated with complicated pregnancy, microRNA (or other noncoding RNAs) content within exosomes could be profiled and used as biomarkers for gestational diseases such as GDM.

## 6. Conclusion and Future Perspectives

Circulating microRNAs have been proposed as potential diagnostic, prognostic, and therapeutic biomarkers of several diseases, being potentially secreted in biological fluids by virtually all cell types [88]. Furthermore, they have been suggested as mediators of tissue cross talk, both in physiological and in pathological conditions [89]. Therefore, the characterization of microRNA expression pattern may also reveal new pathogenic mechanisms, thus improving our understanding of several diseases. Clearly, there is a paucity of data regarding the expression of circulating serum/plasma microRNAs in GDM. The results of the studies reported above highlight a large number of potential candidate circulating biomarkers for GDM. Although informative, the data are discordant, probably due to the different samples analyzed (serum versus plasma) and to the different processing protocols used.

As for the choice between serum or plasma, recent evidence suggests the use of plasma over serum to avoid biases linked to coagulation: as a matter of fact, during this process, microRNAs are released from intact cells or platelets, possibly altering the subsequent results [13, 90]. In other diseases involving different alterations, it may be worthwhile to analyze miRNA profiles in serum over plasma [13, 91, 92].

Another important issue to be considered is the lack of global accepted and standardized operating procedures for sample processing, quality control evaluation, RNA extraction, microRNA profiling method, and data analysis [93]. Therefore, a strong scientific community effort in standardizing common protocols to definitely analyze circulating noncoding RNAs is undoubtedly needed. A step forward is the advancement of novel analytical methodologies. The recently introduced technologies based on next-generation sequencing (NGS) approaches for the evaluation of RNAs represent a novel tool to render circulating microRNA analysis more precise and powerful in terms of absolute quantification and RNA identification. In addition, several novel cDNA library preparation chemistries for small RNA analysis have been recently developed; such methods allow the preparation of complete and highly representative libraries from very small amount of plasma RNA (from 1 ng), thus opening to the possibility to work with low input volume of starting plasma. Finally, NGS technologies allow also the identification of novel classes of small RNAs (e.g., piRNA, tRNA fragments) which may represent additional disease biomarkers in order to be added to the plethora of potential interesting target.

Additionally, the analysis of noncoding RNA content of circulating exosomes represents another aspect to be carefully taken into consideration for future liquid biopsy approaches. Indeed, it is now possible to isolate circulating exosomes derived from a specific cell source, following to an adequate identification of a unique, distinguishing tissue/cell transmembrane protein marker which characterizes that exosomal population [94]. Specifically, as for placenta-derived exosomes, the transmembrane enzyme PLAP (placental alkaline phosphatase), identified as placenta-specific marker, could be potentially used to immunocapture circulating exosomes derived from placenta thus specifically allowing the analysis of their content [95, 96].

Surely, microRNAs represent potential biomarkers for early GDM diagnosis, and to comprehend its pathogenic mechanisms, however, additional studies are necessary to grasp the physiological and pathological patterns of expression of these molecules in pregnancy. Moreover, the characterization of standard operating procedures (SOPs) to collect serum or plasma, to extract RNA, to measure circulating microRNAs, and to analyze their expression profile is required to achieve this important goal.

## Figures and Tables

**Table 1:** The results reported by several studies on circulating microRNAs and GDM.

Study	Study design	Source	Method employed (statistical analysis)	Upregulated microRNA in GDM	Downregulated microRNA in GDM	Limits of the study
Zhao et al. [51]	24 GDM patients and 24 controls at 16th–19th week of pregnancy	Serum	TaqMan low-density array qRT-PCR (Student's *t*-test + risk score analysis)	/	miR-29a, miR-123, miR-222	Normalization using only spiked synthetic exogenous cel-miR-39

Zhu et al. [55]	10 GDM patients and 10 controls at 16th–19th week of pregnancy	Plasma	Ion Torrent qRT-PCR (Student's *t*-test)	miR-16-5p, miR-17-5p, miR-19a-3p, miR-19b-3p, miR-20a-5p	/	Identified microRNAs are enriched in erythrocytes (limitation for their use as biomarkers)

Cao et al. [56]	85 GDM patients and 72 controls at 16th–20th, 20th–24th, and 24th–28th week of pregnancy	Plasma	qRT-PCR (Student's *t*-test, ROC curves)	miR-16-5p, miR-17-5p, miR-20a-5p	/	Identified microRNAs are enriched in erythrocytes (limitation for their use as biomarkers)

Wander et al. [65]	36 GDM patients and 80 controls at 7th–23rd week of pregnancy	Plasma	qRT-PCR (logistic regression adjusted for gestational age at blood draw)	miR-155-5p, miR-21-3p, miR-210-3p, miR-155-5p, miR-146b-5p, miR-223-3p, miR-517-5p, miR-29a-3p	/	Limited number of microRNAs analyzed (associated with pregnancy course and complications)

Lamadrid-Romero et al. [69]	54 GDM patients, 57 non-GDM controls at first, second, and third trimesters of pregnancy, 10 nonpregnant healthy women	Serum	qRT-PCR (Turkey's multiple comparison test + unpaired Student's *t*-test)	miR-183-5p, miR-200b-3p, miR-125-5p, miR-1290	/	Limited number of microRNAs analyzed (neural development associated)

Sebastiani et al. [13]	21 GDM patients, 11 non-GDM control subjects (28th–33rd week of pregnancy)	Plasma	Stem-loop RT PCR (Student's *t*-test + nonparametric Mann–Whitney *U* test)	miR-330-3p	/	To be validated in a larger cohort
